# Prenatal oral health guidelines: a theory- and practice-informed approach to survey development using a modified-Delphi technique and cognitive interviews

**DOI:** 10.1186/s43058-022-00363-6

**Published:** 2022-11-28

**Authors:** Cheryl A. Vamos, Stacey B. Griner, Ellen M. Daley, Morgan Richardson Cayama, Jason Beckstead, Kim Boggess, Rocio B. Quinonez, Laura Damschroder

**Affiliations:** 1 College of Public Health, University of South Florida, 13201 Bruce B. Downs Blvd MDC 56, Tampa, FL 33612 USA; 2 School of Public Health, The University of North Texas Health Science Center at Fort Worth, 3500 Camp Bowie Blvd, Fort Worth, TX 76107 USA; 3 Department of Obstetrics and Gynecology, School of Medicine, University of North, Carolina at Chapel Hill, CB 7516, Chapel Hill, NC 27599 USA; 4 Department of Pediatric Dentistry, Schools of Dentistry, Pediatrics and Public Health, University of North Carolina at Chapel Hill, CB 7450, Chapel Hill, NC 27599 USA; 5 Implementation Pathways, Chelsea, Michigan USA

**Keywords:** Consolidated Framework for Implementation Research, Prenatal oral health, Survey design

## Abstract

**Background:**

Pregnancy presents an opportune time for oral health promotion and intervention; however, implementation of the prenatal oral health guidelines remains a challenge among prenatal and oral health providers. The purpose of this study was twofold: To employ a theory-based approach to identify high-priority Consolidated Framework for Implementation Research (CFIR) constructs with the greatest potential to impact prenatal oral health guideline implementation, and to operationalize and pre-test survey items based on the prioritized CFIR constructs. Identifying barriers and facilitators to guideline implementation will inform the development of targeted interventions that address gaps in adherence which can positively impact oral-systemic health.

**Methods:**

The online survey development process employed three rounds of a modified-Delphi technique with prenatal (i.e., MD/DO, CNM) and oral health (i.e., DMD) Practice Advisory Board Members, cognitive interviews with prenatal and oral health providers, and deliberations among the research team and a Scientific Advisory Board (OBGYN, pediatric dentist, and researchers). High-impact CFIR constructs were identified and translated into survey items that were subsequently piloted and finalized.

**Results:**

During three modified-Delphi rounds, a total of 39 CFIR constructs were evaluated with final input and deliberations with the Practice Advisory Board, Scientific Advisory Board, and the research team achieving consensus on 19 constructs. The instrument was pre-tested with four prenatal and two oral health providers. Overall, participants reported that the survey items were feasible to respond to, took an appropriate length of time to complete, and were well-organized. Participants identified specific areas of improvement to clarify CFIR items. The final survey instrument included 21 CFIR items across four domains, with five constructs included from the intervention characteristics domain, two from the process domain, two from the outer setting domain, and 12 from the inner setting domain.

**Conclusions:**

Lessons learned from the survey development process include the importance of soliciting diverse scientific and practice-based input, distinguishing between importance/impact and direction of impact (barrier/facilitator), and the need for additional qualitative methods during interdisciplinary collaborations. Overall, this study illustrated an iterative approach to identifying high-priority CFIR constructs that may influence the implementation of the prenatal oral health guidelines into practice settings.

Contributions to the literature

- This study uses the meta-framework Consolidated Framework for Implementation Research (CFIR) to devise a theory-driven survey on barriers and facilitators to prenatal oral health guideline implementation.
- The final survey consisted of 21 items that were derived from 19 of the 39 CFIR constructs and was overall deemed highly acceptable among prenatal and oral health provider pilot participants.
- This study provides lessons learned in the theory-based survey development process that can prove useful to researchers and practitioners seeking to measure drivers of evidence-based guideline implementation within clinical settings.

## Background

Oral health during pregnancy is a significant concern given the prevalence of poor oral health conditions (e.g., gingivitis, periodontal disease), gaps in oral healthcare, and associations between oral-systemic health including adverse pregnancy and birth outcomes (e.g., preterm birth), early childhood caries, and other chronic conditions [1–6]. Pregnancy presents an opportune time for oral health promotion and intervention that can impact the health of mothers and children [7, 8]. Subsequently, a national consolidated set of prenatal oral health guidelines was co*-*endorsed by both the American College of Obstetricians and Gynecologists and the American Dental Association [9] (Table 1). Nonetheless, guideline implementation into practice remains a challenge. Research suggests an ongoing lack of guideline awareness, implementation, and interprofessional coordination among prenatal providers and oral healthcare providers [10, 11].

**Table 1:** Select oral health guideline behaviors for prenatal and oral health providers

Guideline recommendation	Prenatal providers	Oral health providers
Assess oral health status	• Take an oral health history • Check the mouth for problems • Document oral health findings in the patient chart	• Take an oral health history • Perform a comprehensive oral examination
Advise about oral health care	• Reassure the patient that oral health care is safe during pregnancy • Encourage the patient to seek oral health care and practice oral hygiene	• Reassure the patient that oral health care is safe during pregnancy • Encourage the patient to seek oral health care and practice oral hygiene
Improve access to oral health services	• Include questions about oral health on patient intake forms • Include oral health in prenatal education	• Accept pregnant patients with Medicaid/public insurance • Establish partnerships with community programs serving pregnant people
Work in collaboration with other health professionals	• Establish relationships with oral health professionals and develop a formal referral process • Share information and coordinate care	• Establish relationships with prenatal providers and develop a formal referral process • Consult with prenatal providers as needed on treatment options, co-morbid conditions, etc.

Effective guideline implementation to improve healthcare quality and outcomes is a national priority [12, 13]. Barriers to guideline implementation are multi-factorial and implementation science frameworks are useful for understanding the extent to which guidelines are adopted and implemented [14, 15]. The Consolidated Framework for Implementation Research (CFIR) is a “meta-theoretical framework” created from a synthesis of 19 pre-existing theories and can guide assessments of implementation barriers and facilitators [16, 17]. The CFIR identifies five domains, consisting of total of 39 individual constructs, that influence implementation: (1) *intervention characteristics* (e.g., quality of evidence; relative advantage), (2) *characteristics of individuals* (e.g., knowledge, self-efficacy), (3) *inner setting* (e.g., culture, networks), (4) *outer setting* (e.g., peer pressure, external policy), and (5) *process* (e.g., planning, executing) [16]. It is recommended that researchers assess the importance of each construct and provide justification for those identified, yet few researchers describe this process [16, 17].

In a systematic review on CFIR application, only three studies provided a rationale, only one study reported working with their population of interest and using all 39 constructs, and none of the studies used quantitative methods [17–20]. Few studies describe the development of quantitative CFIR measures [21–23]. However, such measures have been developed for use during or after the implementation, using a retrospective identification of barriers and facilitators [23–25]. There is a need to apply the CFIR prospectively, before or early in the implementation process, to identify salient factors to inform implementation strategies [17].

The purpose of this study was to employ a quantitative, theory-based yet practical approach to identifying CFIR constructs with the highest potential to impact implementation. A secondary purpose was to operationalize, draft, and pre-test survey items based on the prioritized CFIR constructs. Lessons learned when balancing survey development with practical consideration are highlighted. Future research will use identified constructs to assess their impact on prenatal oral health practice behaviors.

## Methods

**Fig. 1:**
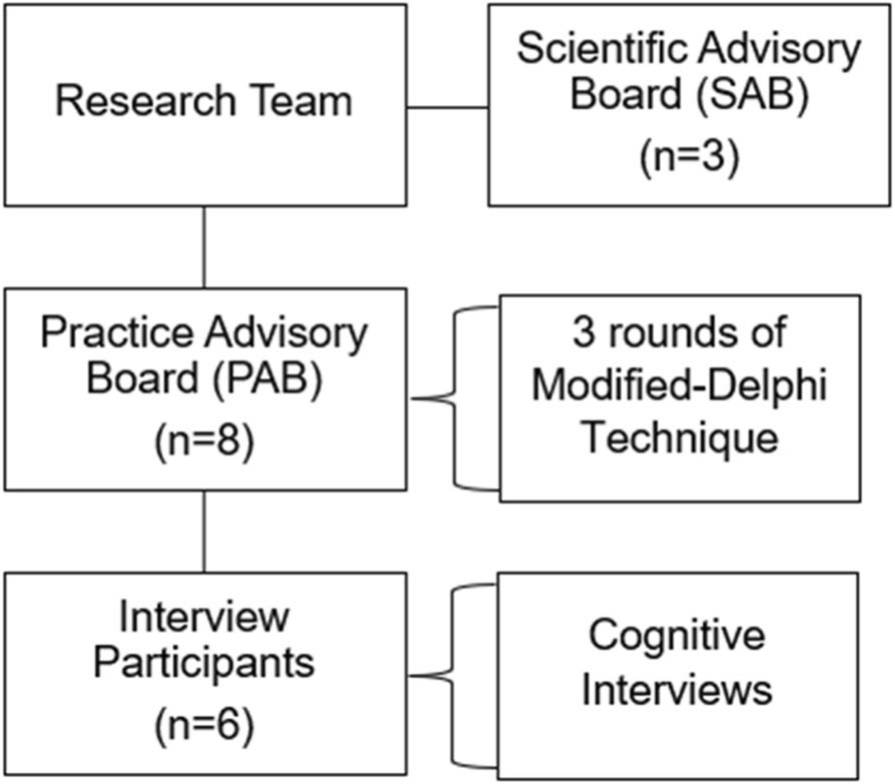
Stakeholder groups involved in the study

This theory-informed survey development process involved collaboration with Practice Advisory Board Members (PAB) during multiple rounds of a modified-Delphi technique, cognitive interviews with prenatal and oral health providers, and deliberations among the research team and a Scientific Advisory Board (SAB). The development process included identifying high-impact CFIR constructs, creating survey items from priority constructs, and piloting and finalizing survey items to be administered to providers in a future study phase. PAB members were recruited via convenience sample from professional prenatal and oral health networks (*n*=8; 2 MD; 2 DO; 2 CNM; 2 DMD/DDS). The SAB included (1) a board-certified obstetrician and maternal-fetal medicine specialist focused on oral disease and pregnancy outcomes, (2) a pediatric dentist specializing in prenatal and early childhood oral health, and (3) a researcher specializing in implementation science and the CFIR. The research team included three public health researchers proficient in oral health, maternal and child health, and survey design/psychometrics. The team adhered to the Checklist for Reporting Results of Internet E-Surveys (CHERRIES) [26]. A graphic illustrating the role of the stakeholder groups is presented in Fig. 1.

### Modified-Delphi technique

The research team developed an online questionnaire based on all CFIR constructs (*n*=39), with input from the SAB. A modified-Delphi process was used with three rounds of surveys administered to the PAB [27–30]. For each round, PAB members individually ranked CFIR constructs using Likert scales to prioritize factors perceived to be most salient (of high priority). Each round utilized a different approach to determine consensus, a process informed by systematic and practical considerations as the rounds progressed and challenges were encountered. The modified-Delphi process is presented in Table 2.

**Table 2:** Modified-Delphi process

	Item stem	Response options	Construct presentation and example	Number of constructs presented in survey round	Consensus definition
Round 1	Please indicate how important you believe each factor may be in influencing whether prenatal/oral health providers implement the guidelines into their daily clinical practice by selecting one of the response options for each factor.	Five-point Likert scale: Not at all important, not very important, neutral, important, very important	Construct definition Intervention source: individuals’ perception about whether the guidelines are externally or internally developed.	39	Greater than or equal to 70% of participant responses falling within two consecutive categories on the Likert scale (e.g., not at all important or not very important, and important or very important).
Round 2	How strong of an impact do you think each of the following factors will have on whether providers implement the prenatal oral health guidelines into their daily clinical practice?	Four-point scale: no impact, weak impact, moderate impact, strong impact.	Translated construct with context Whether the guidelines are externally (e.g., ACOG, ADA) or internally (e.g., within your clinic) developed.	39	Moderate to strong impact, a mean ranking greater than or equal to 2.0.
Round 3	Same as Round 2	Same as Round 2	Same as Round 2	17 (only those with no consensus or mixed results from Round 2)	Same as Round 2

#### Round 1

All 39 constructs and their definitions were presented, with one item representing each construct. Participants were asked to indicate how important they believe each factor may be in influencing whether providers implement the guidelines into clinical practice. The response options were provided on a 5-point Likert scale ranging from (1) *not at all important* to (5) *very important*. Consensus was defined as ≥70% of responses falling within two consecutive categories (e.g., not at all important/not very important; important/very important) [27, 29, 31]. Prior to the survey, the PAB was provided with information on the guidelines and a brief introduction to the CFIR. Definitions provided for each CFIR construct were *not* operationalized or translated to prenatal oral health but were defined directly from CFIR guidance [32].

#### Round 2

Most constructs were rated as very important/important by the PAB in Round 1. The questionnaire was revised based on discussions between researchers and the SAB to address challenges with consensus and item wording. Revisions included the following: (1) Question stem was changed from *importance* to *impact* to reduce a bias toward inclusion and was modified to: How strong of an impact do you think each of the following factors will have on whether providers implement the prenatal oral health guidelines into their daily clinical practice?; (2) Items were modified to a translated, operationalized version to facilitate readability and application among providers; (3) Response options were changed to a four-point scale ranging from (1) *no impact* to (4) *strong impact*; and (4) Definition of consensus was changed; consensus was considered reached when a construct had a mean impact of ≥2.0 (moderate/strong impact). All 39 CFIR constructs were included in this round.

#### Round 3

This round included *only* those constructs without consensus or those with mixed results from earlier rounds. The purpose was to determine if consensus could be achieved when updated items from Round 2 were presented to PAB members.

Overall, while the intention of the modified-Delphi process was to narrow down constructs in a clear, linear manner across rounds, we encountered practical challenges where PAB members felt that most constructs were important to include and/or there was no consensus on constructs to be retained. These challenges may have been due to the lack of operationalization and translation of the CFIR constructs in Round 1, requiring revision of all CFIR constructs in Round 2.

### Cognitive interviews

After the instrument was revised based on feedback from the SAB and modified-Delphi rounds with the PAB, cognitive interviews were conducted to assess content validity, feasibility, and acceptability among a convenience sample of prenatal and oral health providers. Inclusion criteria were as follows: (1) ≥21 years old, (2) licensed healthcare professional (DMD, MD/DO, CNM), and (3) currently provide prenatal or oral healthcare to pregnant patients. Six participants were recruited through the PAB and the SAB, an appropriate sample size for cognitive interviews using this methodology [33]. Participants completed the online survey and were emailed a copy to review during the interview with the research team.

The cognitive interview guide included questions assessing participants’ overall thoughts of the survey, flow, length, content, readability, and extent of ambiguity of the items and response options. A verbal probing approach was used and elicited insight on understanding, survey content, and organization [33]. The interviews lasted approximately 45 min and detailed notes were recorded. Each participant received $100 for their time. Findings were reviewed and items that were unclear, double-barreled, or misunderstood were revised or removed.

## Results

### Identifying High-Priority Constructs

In Round 1 of the modified-Delphi process, 24 of the 39 CFIR constructs had consensus based on ≥70% of responses spread across two consecutive categories. In Round 2, after presenting all 39 constructs and using modified question stems and response options, 20 constructs reached consensus. In Round 3, the remaining 17 constructs that did not reach consensus in the prior two rounds were presented and assessed using the same consensus definition from Round 2. A total of 14 constructs reached consensus after this round, but the consensus process and construct retainment were revisited as explained below.

### Final deliberation on achieving consensus

In reviewing all three rounds and discussing the various approaches and language to survey items, it was decided to re-analyze Round 2 data (which included the operationalized 39 CFIR constructs) using a modified cut-off value calculated based on Round 2’s mean response across all constructs. For each of the five CFIR domains, item means were ranked ordered (from most to least important), and constructs with the highest mean rankings within each domain were retained. This calculation resulted in 19 retained constructs prior to SAB, PAB, and cognitive interview feedback. The SAB and PAB reviewed the final instrument to ensure that items were clear, scientifically relevant, and had utility to practice settings.

### Pre-testing through cognitive interviews

The instrument with items translated from the retained CFIR constructs was pre-tested with six participants (4 prenatal and 2 oral health providers). The survey took an average of 13 min to complete and sociodemographic characteristics of cognitive interview participants are provided in Table 3.

**Table 3:** Socio-demographic characteristics of cognitive interview participants (*n*=6)

Participant ID	Degree	Gender	Race	Ethnicity	Length of survey
CI1	MD, MPH	F	White	Not Hispanic	7:44
CI2	DMD	F	White	Hispanic	19:24
CI3	CNM	F	White	Not Hispanic	7:28
CI4	DMD	M	Black	Not Hispanic	13:40
CI5	MD, MPH	F	White	Not Hispanic	5:18
CI6	CNM	F	White	Not Hispanic	25:24

### Feedback from cognitive interviews

#### General modifications

Participants reported that survey items were acceptable and feasible for providers and were well organized within the survey. Participants suggested adding clarifying information at the beginning of the survey to advise participants to not look up information about the guidelines prior to their participation as they would receive more information later in the survey. Participants also recommended adding “I don’t know” as a response option regarding guideline awareness, and changing the wording of the stem for CFIR items to: When *deciding to implement* the prenatal oral health guidelines into practice, how *important* are the following things?

#### CFIR construct changes

Participants identified several areas of improvement in clarifying CFIR items (Table 4). Two items in particular, *champions* and *formally appointed opinion leaders*, were perceived as unclear because these roles often overlap in practice. One interviewee described the difference between the two constructs: “A champion is someone who is excited to do it or a colleague, a formally appointed opinion leader was someone who was higher up and yelled at you to do it”*.* After soliciting SAB and PAB feedback on these items, the champion item was expanded to include the definition of a champion as “a colleague who takes it upon themselves to promote and support the guidelines”.

**Table 4:** Changes to CFIR survey items after cognitive interviews

CFIR construct	Initial survey item	Final survey item, post-edits
Implementation Climate	The degree to which my organization expects and supports the implementation of the guidelines.	Changed to two items: The degree to which my organization **expects** the implementation of the guidelines The degree to which my organization **supports** the implementation of the guidelines.
	The compatibility of the guidelines with the workflow of my organization.	The **compatibility** of the guidelines to fit within the current workflow of my organization.
	The degree to which feedback on implementation progress is provided and acted upon in my organization.	The degree to which my organization **provides feedback** on the implementation process.
Implementation Readiness	The degree to which there are dedicated resources for implementing the guidelines into my organization (e.g., money, training, time).	The degree to which there are **dedicated resources** for implementing the guidelines into my organization (e.g., staff, money, training, time).
	The degree to which there is access to easy digestible information about the guidelines and how to implement them into practice.	The degree to which there is **access to information** about the guidelines and **how** to implement them into practice.
Process	Having a champion in my organization to support and reinforce the guidelines.	Having a **champion** (e.g., someone who takes it upon themselves to promote and support the guidelines) in my organization.

Similarly, there were challenges differentiating between *compatibility* and *adaptability*. One participant felt that “Adaptability and compatibility with the workflow are the same – if it’s adaptable, you can change it to be compatible with your workflow. I might make the difference between the two more clear – one is more like changing the guidelines, one like changing your part in it.” Based on this feedback, these items were modified to clarify the distinct constructs.

### Final instrument

The final survey instrument included 21 CFIR items across four domains after separating double-barreled items; implementation climate and leadership engagement were measured by two items each. Five constructs were included from the *intervention characteristics* domain, two from the *process* domain, two from the *outer setting* domain, and 12 from the *inner setting* domain. Table 5 includes the final operationalization of prioritized CFIR constructs.

**Table 5:** Final survey

**Introduction to survey**	In 2012, a national expert panel comprehensively reviewed the evidence, assessed existing guidelines, and synthesized key recommendations for both prenatal and oral health providers. This led to the development of **Oral Health Care during Pregnancy: A National Consensus Statement**, endorsed by both the American College of Obstetricians and Gynecologists (ACOG) and the American Dental Association (ADA). The guidelines highlight the following overarching practice behaviors for both prenatal and oral health providers with regards to oral health among their pregnant patients: (1) assess, (2) advise, and (3) refer and coordinate care. **• Assess:** Examples include taking an oral health history; checking the mouth for problems; documenting the findings. **• Advise:** Examples include encouraging women to seek oral health care; counseling women on good oral hygiene behaviors. **• Refer and coordinate care:** Examples include referring women to prenatal/oral health providers; collaborating and engaging in interprofessional care. The next set of questions ask you to think about factors that may influence your decision to implement the prenatal oral health guidelines into daily practice.	
**Item stem**	How **important** are the following factors to you when **deciding to implement** the prenatal oral health guidelines into your daily practice?	
**CFIR domain**	**Construct**	**Survey item**
Intervention characteristics	Evidence strength and quality	The **strength of evidence** to support the guidelines.
	Adaptability	The **adaptability** of the guidelines to meet my organization’s needs.
	Design quality and packaging	The **presentation and packaging** of the guidelines for dissemination.
	Complexity	The **complexity** of the guidelines.
	Cost	The **costs** associated with implementing the guidelines.
Process	Formally appointed opinion leaders	Having a **formally appointed implementation leader** in my organization for these guidelines.
	Champions	Having a **champion** (e.g., someone who takes it upon themselves to promote and support the guidelines) in my organization.
Outer setting	Patient needs and resources	The degree to which the guidelines **address the needs of my patients**.
	Cosmopolitanism	The degree to which my organization **has connections with other [*prenatal/oral health]*providers**.*
Inner setting	Implementation climate	The degree to which **my organization expects** the implementation of the guidelines.
	Implementation climate	The degree to which **my organization supports** the implementation of the guidelines.
	Tension for change	The degree to which my organization feels that the **current oral health care practices** (i.e., prevention, screening, treatment) among pregnant women are **inadequate and need to change.**
	Compatibility	The **compatibility** of the guidelines to fit within the **current workflow** of my organization.
	Relative priority	The **priority** placed on implementing the guidelines relative to other activities.
	Goals and feedback	The degree to which the guidelines **align with my organization’s goals**.
	Goals and feedback	The degree to which my organization provides **feedback** on the implementation process.
	Learning Climate	The degree to which my organization allows me to **test, reflect on, and evaluate** implementation of the guidelines.
	Leadership engagement	The level of **commitment** that my organization has to implement the guidelines.
	Leadership engagement	The degree to which the guidelines are **supported by leaders** within my organization.
	Available resources	The degree to which there are **dedicated resources** for implementing the guidelines into my organization (e.g., staff, money, training, time).
	Access to knowledge and information	The degree to which there is **access to information** about the guidelines and **how** to implement them into practice.

*Note: Item(s) indicated by an asterisks in the table will state either prenatal or oral health provider depending on the participant provider type responding to that survey

## Discussion

This study describes a multi-step process to developing a theory-based and practice-informed survey to identify context-specific barriers and facilitators influencing prenatal oral health guideline implementation. This process used three rounds of a modified-Delphi technique, followed by cognitive interviews, and deliberations among the research team, SAB, and PAB members to identify, operationalize, and pre-test CFIR survey items. Many studies using the CFIR to implement and evaluate clinical practices rely largely on qualitative methods to identify salient constructs [34–39]. Our approach is unique in employing an interactive process that begins with examining all CFIR constructs to prospectively and quantitatively examine implementation barriers and facilitators [15, 17].

A key finding is the significant challenge researchers encounter when identifying high-priority constructs. It is vital to emphasize to stakeholders that the goal is to identify only those factors of the *highest* priority. It may be helpful to clarify at this stage that the task is not to classify the directionality of the factor (i.e., barrier vs. facilitator), but to identify all factors that may have an impact on implementation. Similarly, researchers should adhere to psychometric principles, including the language of items and other measurement issues, to facilitate readability and data analysis, production of cut-off values, and decisions about consensus.

The process of achieving consensus can be difficult. Ideally, researchers should establish how consensus will be operationalized and statistically analyzed prior to the study. Nonetheless, given that implementation can be complex in practice, quantitative approaches to measuring implementation constructs must be flexible. In this study, the PAB was emailed instructions, the rationale for this process, and a link to complete the survey for each round. This passive, one-directional communication process did not permit an interactive dialog where constructs could be discussed before completing the survey [27, 28, 30]. Future research should consider including a group forum to facilitate discussion, generation of meaning-making, and opportunity for participants to consider varying perspectives.

Pre-testing the survey via cognitive interviews is paramount to improve survey quality (e.g., clarity, flow) [33, 40–42]. The pre-testing process can illuminate how target participants think and behave in research and practice. This phase also underscores the need to utilize health literacy principles, such as explaining what is being asked of them and why, using laymen’s terms, and avoiding double-barreled questions and response options.

Study findings must be considered in light of limitations. A modified-Delphi technique was used to establish consensus instead of a traditional linear Delphi technique method with strict processes and cut-off points. The lack of racial/ethnic diversity and professional roles and use of convenience sampling to recruit participants limits generalizability. The modified-Delphi process could have benefitted from additional participants and methods, such as focus groups, to capture diverse perspectives, including those working across a range of practice settings and holding various positions. Nonetheless, this study’s strengths include an interdisciplinary research team and SAB and PAB members, which comprised stakeholders involved in implementation science, MCH oral health guideline implementation, and healthcare delivery. This study highlights the need for both scientific rigor and flexibility when systematically identifying, translating, and prioritizing theoretical constructs to real-world practice.

## Conclusions

Our process and lessons learned may be useful to others who are applying an implementation science theoretical framework, such as CFIR, to develop a quantitative survey to identify barriers and facilitators to implementing guidelines in other settings. Key lessons learned include the importance of soliciting diverse input, distinguishing between importance/impact and direction of impact, and need for additional qualitative methods to elicit context and consensus-building. Balancing theoretical and practical applications remains critical while identifying high-priority implementation factors and advancing theoretical contributions to implementation science.

## Data Availability

The datasets used and/or analyzed during the current study are available from the corresponding author on reasonable request.

## Abbreviations

CFIR: Consolidated Framework for Implementation Research
PAB: Practice Advisory Board
SAB: Scientific Advisory Board
